# Methyl 2-[2-(benzyl­oxycarbonyl­amino)­propan-2-yl]-5-hy­droxy-6-meth­oxy­pyrimidine-4-carboxyl­ate

**DOI:** 10.1107/S160053681005467X

**Published:** 2011-01-08

**Authors:** Hoong-Kun Fun, V. Sumangala, D. Jagadeesh Prasad, Boja Poojary, Suchada Chantrapromma

**Affiliations:** a X-ray Crystallography Unit, School of Physics, Universiti Sains Malaysia, 11800 USM, Penang, Malaysia; b Department of Chemistry, Mangalore University, Mangalagangotri 574 199, Karnatak State, India; c Crystal Materials Research Unit, Department of Chemistry, Faculty of Science, Prince of Songkla University, Hat-Yai, Songkhla 90112, Thailand

## Abstract

In the title compound, C_18_H_21_N_3_O_6_, a pyrimidine derivative, the dihedral angle between the benzene and pyrimidine rings is 52.26 (12)°. The carboxyl­ate unit is twisted with respect to the pyrimidine ring, making a dihedral angle of 12.33 (7)°. In the crystal, mol­ecules are linked by a pair of O—H⋯O hydrogen bonds, forming an inversion dimer. The dimers are stacked into columns along the *b* axis through weak C—H⋯O inter­actions.

## Related literature

For bond-length data, see: Allen *et al.* (1987 ▶). For hydrogen-bond motifs, see: Bernstein *et al.* (1995 ▶). For background to and applications of pyrimidine derivatives, see: Cheng & Roth (1971 ▶); Cox (1968 ▶); Eussell (1945 ▶); Jain *et al.* (2006 ▶); Shinogi (1959 ▶); Tani *et al.* (1979 ▶). 
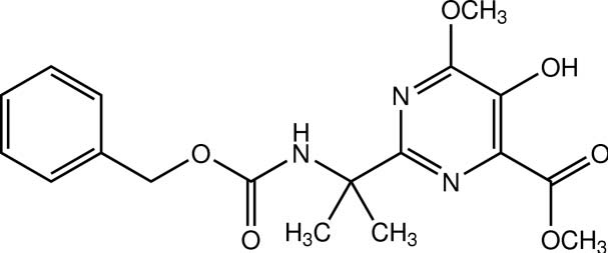


## Experimental

### 

#### Crystal data

C_18_H_21_N_3_O_6_
*M* *_r_* = 375.38
Monoclinic, 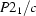

*a* = 16.5226 (2) Å
*b* = 8.5717 (1) Å
*c* = 13.0944 (2) Å
β = 97.236 (1)°
*V* = 1839.75 (4) Å^3^
*Z* = 4
Mo *K*α radiation
μ = 0.10 mm^−1^
*T* = 297 K
0.57 × 0.52 × 0.39 mm

#### Data collection

Bruker APEXII CCD area-detector diffractometer
Absorption correction: multi-scan (*SADABS*; Bruker, 2005 ▶) *T*_min_ = 0.944, *T*_max_ = 0.961
20111 measured reflections
5348 independent reflections
4087 reflections with *I* > 2σ(*I*)
*R*_int_ = 0.024

#### Refinement

*R*[*F* ^2^ > 2σ(*F* ^2^)] = 0.054
*wR*(*F* ^2^) = 0.164
*S* = 1.04
5348 reflections
256 parameters
H atoms treated by a mixture of independent and constrained refinement
Δρ_max_ = 0.52 e Å^−3^
Δρ_min_ = −0.33 e Å^−3^

### 

Data collection: *APEX2* (Bruker, 2005 ▶); cell refinement: *SAINT* (Bruker, 2005 ▶); data reduction: *SAINT*; program(s) used to solve structure: *SHELXTL* (Sheldrick, 2008 ▶); program(s) used to refine structure: *SHELXTL*; molecular graphics: *SHELXTL*; software used to prepare material for publication: *SHELXTL* and *PLATON* (Spek, 2009 ▶).

## Supplementary Material

Crystal structure: contains datablocks global, I. DOI: 10.1107/S160053681005467X/is2653sup1.cif

Structure factors: contains datablocks I. DOI: 10.1107/S160053681005467X/is2653Isup2.hkl

Additional supplementary materials:  crystallographic information; 3D view; checkCIF report

## Figures and Tables

**Table 1:** Hydrogen-bond geometry (Å, °)

*D*—H⋯*A*	*D*—H	H⋯*A*	*D*⋯*A*	*D*—H⋯*A*
O4—H1*O*4⋯O5	0.87 (2)	1.93 (3)	2.6513 (15)	139 (2)
O4—H1*O*4⋯O2^i^	0.87 (2)	2.39 (2)	3.0508 (17)	132 (2)
C7—H7*A*⋯O5^i^	0.97	2.52	3.347 (3)	143
C15—H15*A*⋯O3^ii^	0.96	2.49	3.2148 (18)	132
C17—H17*A*⋯O2	0.96	2.54	3.099 (2)	117

Symmetry codes: (i) 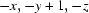; (ii) 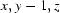.

**Fig. 1.**
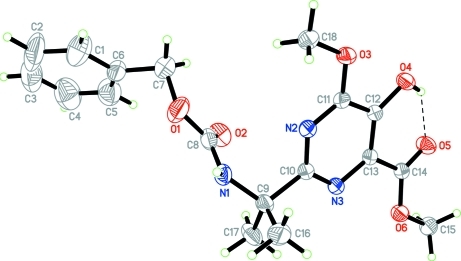
The molecular structure of the title compound, showing 40% probability displacement ellipsoids and the atom-numbering scheme. Hydrogen bond is shown as dashed line.

**Fig. 2.**
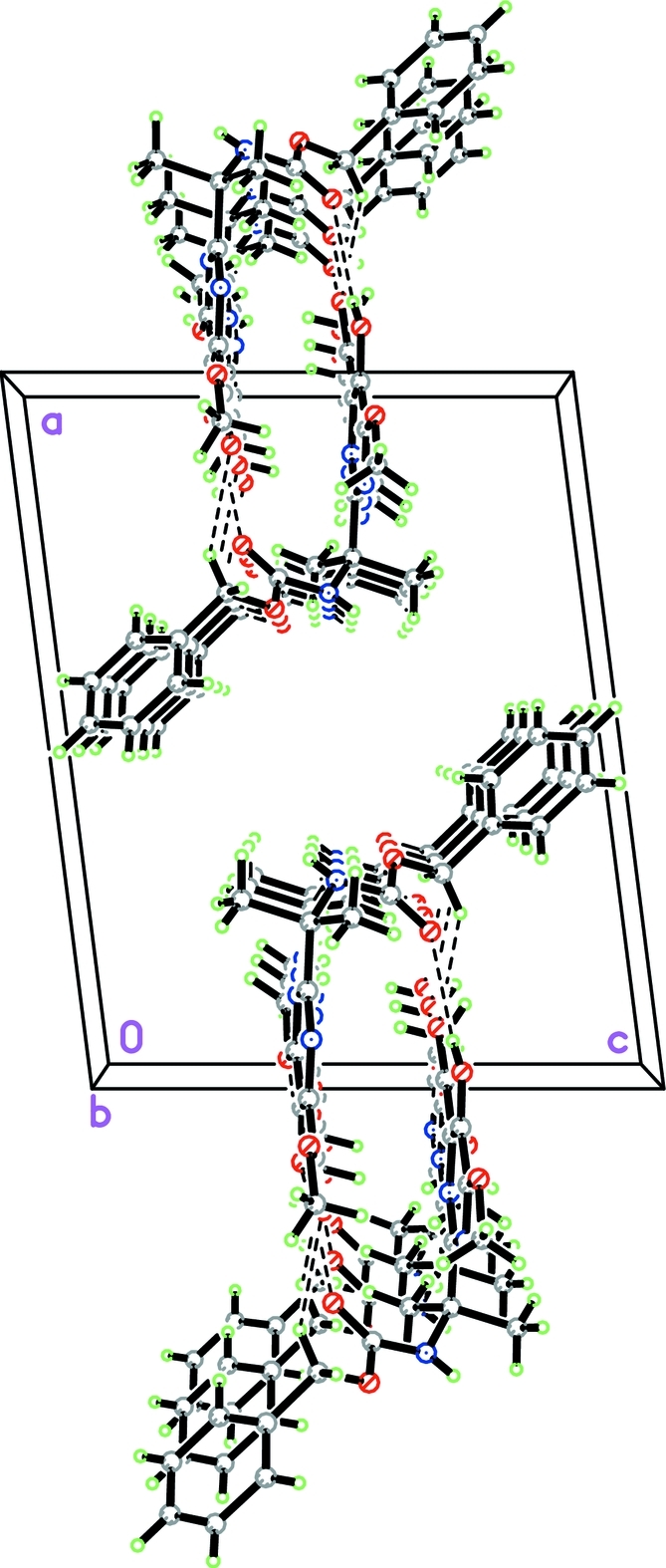
The crystal packing of the title compound viewed along the b axis, showing columns along the b axis. Hydrogen bonds are shown as dashed lines.

## supplementary crystallographic information

### Comment

Several pyrimidine derivatives have been developed as chemotherapeutic agents and have found wide clinical applications (Jain *et al.*, 2006). The pyrimidine ring is found in vitamins like thiamine, riboflavin and folic acid (Cox, 1968). Barbiton, a pyrimidine derivative, possesses hypnotic, sedative and anticonvulsant (Eussell, 1945) activities. Pyrimidine derivatives of sulfa drugs, namely sulfadiazine, sulfamerazine and sulfadimidine are superior to many other sulfonamides and are used for treatment in some acute UT infections, cerebrospinal meningitis and for patients allergic to pencillins (Shinogi, 1959). 2-Thiouracil and its alkyl analogue, thiobarbital, are effective drugs against hyperthyroidism. Propylthiouracil is used as a drug for hyperthyroidism with minimum side effects (Cheng & Roth, 1971). Afloqualone has been evaluated as a successful anti-inflammatory agent with lower-back-pain patients (Tani *et al.*, 1979). In view of the importance of pyrimidine derivatives, the title compound (I) was synthesized and its crystal structure was reported.

The molecule of (I), (Fig. 1), is a V-shaped structure with the dihedral angle between the benzene and pyrimidine rings being 52.26 (12)°. The oxycarbonylamino unit (atoms C8, N1, O1 and O2) are planar with *r.m.s*. 0.0035 (1) Å. This unit makes dihedral angles of 62.35 (12) and 65.98 (8)° with the benzyl group (C1—C7) and pyrimidine ring, respectively. The hydroxy group is co-planar with the pyrimidine ring [*r.m.s*. 0.0190 (1) Å] whereas the methoxy is slightly deviated with the torsion angle C18–O3–C11–C12 = 175.74 (12)°. The carboxylate moiety is planar with *r.m.s*. 0.0033 (1) Å and makes the dihedral angle of 12.33 (7)° with the pyrimidine ring. The conformation of the carboxylate moiety is indicated by the torsion angles of C15–O6–C14–C13 = 179.53 (11)° and C12–C13–C14–O5 = -10.7 (2)°. Intramolecular O4–H1O4···O5 hydrogen bond generate S(6) ring motif (Bernstein *et al.*, 1995). The bond distances are of normal values (Allen *et al.*, 1987).

In the crystal packing (Fig. 2), the molecules are linked bya pair of O—H···O hydrogen bonds (Table 1), forming an inversion dimer. These dimers are stacked into columns along the *b* axis through weak C—H···O interactions (Table 1). The crystal is solidated and stabilized by O—H···O hydrogen bonds and C—H···O weak interactions (Table 1).

### Experimental

The title compound was synthesized by taking benzyl (1-cyano-1-methylethyl) carbamate (0.10 mole) in xylene and dimethyl acetylene dicarboxylate (0.12 mole) was added. The reaction mixture is heated to 407 K and maintained at the temperature for 10 hrs until the reaction was completed. On cooling, the precipitated product, methyl 2-(1-{[(benzyloxy) carbonyl] amino}-1-methylethyl)-5,6-dihydroxypyrimidine-4-carboxylate, is filtered and washed with hexane. The sample is dried at 313 K for 4–5 hrs. The obtained material is taken in 5 volume of methanol and 0.22 mole of 5% sodium hydroxide in methanol. The mixture was cooled to 288 K and methyl iodide (0.20 mole) was then added and heated to 323 K. After the reaction was over, methanol was distilled off and the product was quenched in water and then filtered to get the title compound. Colorless block-shaped single crystals of the title compound suitable for *x*-ray structure determination were recrystalized from ethanol by the slow evaporation of the solvent at room temperature after several days, Mp. 391–393 K.

### Refinement

Amide and hydroxy H atoms are located in a difference map and refined isotropically. The remaining H atoms were positioned geometrically and allowed to ride on their parent atoms, with *d*(C—H) = 0.93 Å for aromatic and 0.96 Å for CH_3_ atoms. The *U*_iso_ values were constrained to be 1.5*U*_eq_ of the carrier atom for methyl H atoms and 1.2*U*_eq_ for the remaining H atoms. A rotating group model was used for the methyl groups.

### Crystal data

**Table:**

C_18_H_21_N_3_O_6_	*F*(000) = 792
*M**_r_* = 375.38	*D*_x_ = 1.355 Mg m^−^^3^
Monoclinic, *P*2_1_/*c*	Melting point = 391–393 K
Hall symbol: -P 2ybc	Mo *K*α radiation, λ = 0.71073 Å
*a* = 16.5226 (2) Å	Cell parameters from 5348 reflections
*b* = 8.5717 (1) Å	θ = 2.5–30.0°
*c* = 13.0944 (2) Å	µ = 0.10 mm^−^^1^
β = 97.236 (1)°	*T* = 297 K
*V* = 1839.75 (4) Å^3^	Block, colorless
*Z* = 4	0.57 × 0.52 × 0.39 mm

### Data collection

**Table:**

Bruker APEXII CCD area-detector diffractometer	5348 independent reflections
Radiation source: sealed tube	4087 reflections with *I* > 2σ(*I*)
graphite	*R*_int_ = 0.024
φ and ω scans	θ_max_ = 30.0°, θ_min_ = 2.5°
Absorption correction: multi-scan (*SADABS*; Bruker, 2005)	*h* = −23→22
*T*_min_ = 0.944, *T*_max_ = 0.961	*k* = −8→12
20111 measured reflections	*l* = −18→18

### Refinement

**Table:**

Refinement on *F*^2^	Primary atom site location: structure-invariant direct methods
Least-squares matrix: full	Secondary atom site location: difference Fourier map
*R*[*F*^2^ > 2σ(*F*^2^)] = 0.054	Hydrogen site location: inferred from neighbouring sites
*wR*(*F*^2^) = 0.164	H atoms treated by a mixture of independent and constrained refinement
*S* = 1.04	*w* = 1/[σ^2^(*F*_o_^2^) + (0.0839*P*)^2^ + 0.5187*P*] where *P* = (*F*_o_^2^ + 2*F*_c_^2^)/3
5348 reflections	(Δ/σ)_max_ = 0.001
256 parameters	Δρ_max_ = 0.52 e Å^−^^3^
0 restraints	Δρ_min_ = −0.33 e Å^−^^3^

### Special details

**Table:**

Geometry. All e.s.d.'s (except the e.s.d. in the dihedral angle between two l.s. planes) are estimated using the full covariance matrix. The cell e.s.d.'s are taken into account individually in the estimation of e.s.d.'s in distances, angles and torsion angles; correlations between e.s.d.'s in cell parameters are only used when they are defined by crystal symmetry. An approximate (isotropic) treatment of cell e.s.d.'s is used for estimating e.s.d.'s involving l.s. planes.
Refinement. Refinement of *F*^2^ against ALL reflections. The weighted *R*-factor *wR* and goodness of fit *S* are based on *F*^2^, conventional *R*-factors *R* are based on *F*, with *F* set to zero for negative *F*^2^. The threshold expression of *F*^2^ > σ(*F*^2^) is used only for calculating *R*-factors(gt) *etc*. and is not relevant to the choice of reflections for refinement. *R*-factors based on *F*^2^ are statistically about twice as large as those based on *F*, and *R*- factors based on ALL data will be even larger.

### Fractional atomic coordinates and isotropic or equivalent isotropic displacement parameters (Å^2^)

**Table:**

	*x*	*y*	*z*	*U*_iso_*/*U*_eq_	
O1	0.33157 (8)	0.66001 (18)	0.07370 (11)	0.0657 (4)	
O2	0.24087 (8)	0.48066 (17)	0.11686 (10)	0.0576 (3)	
O3	0.06818 (6)	0.74259 (11)	−0.14162 (9)	0.0416 (3)	
O4	−0.06047 (6)	0.56031 (12)	−0.13886 (9)	0.0417 (3)	
O5	−0.10852 (6)	0.26969 (13)	−0.11206 (10)	0.0473 (3)	
O6	−0.01903 (6)	0.07581 (11)	−0.11913 (8)	0.0393 (2)	
N1	0.30737 (7)	0.45068 (16)	−0.02407 (11)	0.0407 (3)	
N2	0.15729 (7)	0.53687 (13)	−0.11038 (9)	0.0355 (3)	
N3	0.10782 (7)	0.27843 (13)	−0.09593 (9)	0.0335 (2)	
C1	0.43521 (17)	0.8804 (4)	0.2532 (3)	0.0946 (9)	
H1A	0.4369	0.9541	0.2015	0.114*	
C2	0.49368 (19)	0.8838 (5)	0.3419 (3)	0.1240 (13)	
H2A	0.5334	0.9611	0.3500	0.149*	
C3	0.49077 (19)	0.7724 (5)	0.4147 (3)	0.1109 (12)	
H3A	0.5300	0.7721	0.4723	0.133*	
C4	0.43242 (19)	0.6625 (5)	0.4054 (2)	0.1040 (10)	
H4A	0.4310	0.5876	0.4566	0.125*	
C5	0.37474 (15)	0.6607 (3)	0.3200 (2)	0.0802 (7)	
H5A	0.3342	0.5848	0.3144	0.096*	
C6	0.37579 (10)	0.7669 (2)	0.24422 (15)	0.0554 (4)	
C7	0.31165 (14)	0.7682 (3)	0.15168 (18)	0.0690 (6)	
H7A	0.2593	0.7402	0.1727	0.083*	
H7B	0.3070	0.8727	0.1230	0.083*	
C8	0.28785 (9)	0.5255 (2)	0.06047 (12)	0.0438 (3)	
C9	0.25579 (8)	0.32633 (17)	−0.07422 (12)	0.0402 (3)	
C10	0.16670 (8)	0.38177 (15)	−0.09313 (10)	0.0334 (3)	
C11	0.08266 (8)	0.59052 (15)	−0.12577 (10)	0.0331 (3)	
C12	0.01324 (8)	0.49250 (15)	−0.12617 (10)	0.0319 (3)	
C13	0.03020 (8)	0.33494 (15)	−0.11279 (10)	0.0306 (3)	
C14	−0.03910 (8)	0.22435 (15)	−0.11428 (10)	0.0327 (3)	
C15	−0.08544 (9)	−0.03516 (18)	−0.11987 (13)	0.0428 (3)	
H15A	−0.0688	−0.1342	−0.1443	0.064*	
H15B	−0.1320	0.0021	−0.1645	0.064*	
H15C	−0.0995	−0.0467	−0.0513	0.064*	
C16	0.28446 (11)	0.2973 (3)	−0.17910 (16)	0.0620 (5)	
H16A	0.2781	0.3910	−0.2195	0.093*	
H16B	0.2524	0.2154	−0.2141	0.093*	
H16C	0.3409	0.2672	−0.1696	0.093*	
C17	0.26450 (10)	0.1795 (2)	−0.00690 (18)	0.0621 (5)	
H17A	0.2431	0.1996	0.0567	0.093*	
H17B	0.3211	0.1517	0.0072	0.093*	
H17C	0.2348	0.0953	−0.0424	0.093*	
C18	0.13886 (10)	0.84324 (18)	−0.13387 (14)	0.0459 (4)	
H18A	0.1215	0.9500	−0.1412	0.069*	
H18B	0.1714	0.8175	−0.1873	0.069*	
H18C	0.1706	0.8291	−0.0679	0.069*	
H1N1	0.3337 (13)	0.501 (3)	−0.0634 (16)	0.056 (6)*	
H1O4	−0.0985 (14)	0.492 (3)	−0.1310 (16)	0.061 (6)*	

### Atomic displacement parameters (Å^2^)

**Table:**

	*U*^11^	*U*^22^	*U*^33^	*U*^12^	*U*^13^	*U*^23^
O1	0.0588 (8)	0.0660 (9)	0.0713 (8)	−0.0192 (6)	0.0041 (6)	−0.0211 (7)
O2	0.0530 (7)	0.0649 (8)	0.0557 (7)	0.0042 (6)	0.0103 (6)	0.0081 (6)
O3	0.0391 (5)	0.0268 (5)	0.0573 (6)	−0.0004 (4)	−0.0006 (4)	0.0011 (4)
O4	0.0325 (5)	0.0345 (5)	0.0567 (6)	0.0049 (4)	0.0008 (4)	0.0005 (4)
O5	0.0312 (5)	0.0390 (6)	0.0718 (8)	0.0009 (4)	0.0068 (5)	0.0000 (5)
O6	0.0327 (5)	0.0293 (5)	0.0552 (6)	−0.0027 (4)	0.0033 (4)	0.0006 (4)
N1	0.0304 (6)	0.0433 (7)	0.0474 (7)	−0.0052 (5)	0.0014 (5)	0.0011 (5)
N2	0.0333 (5)	0.0301 (5)	0.0416 (6)	−0.0014 (4)	−0.0004 (4)	−0.0009 (4)
N3	0.0304 (5)	0.0298 (5)	0.0394 (6)	0.0008 (4)	0.0003 (4)	−0.0007 (4)
C1	0.0813 (16)	0.0911 (19)	0.111 (2)	−0.0374 (15)	0.0084 (15)	−0.0063 (16)
C2	0.0691 (17)	0.151 (3)	0.147 (3)	−0.053 (2)	−0.0074 (19)	−0.038 (3)
C3	0.0686 (16)	0.165 (4)	0.093 (2)	0.002 (2)	−0.0162 (15)	−0.040 (2)
C4	0.089 (2)	0.142 (3)	0.0788 (17)	0.013 (2)	0.0018 (14)	0.0011 (18)
C5	0.0657 (13)	0.0840 (17)	0.0898 (16)	−0.0073 (12)	0.0052 (12)	0.0038 (13)
C6	0.0419 (8)	0.0564 (10)	0.0666 (11)	−0.0045 (7)	0.0025 (8)	−0.0181 (9)
C7	0.0615 (11)	0.0586 (11)	0.0818 (14)	0.0012 (9)	−0.0102 (10)	−0.0192 (10)
C8	0.0332 (7)	0.0502 (8)	0.0455 (8)	0.0006 (6)	−0.0050 (6)	0.0024 (6)
C9	0.0293 (6)	0.0348 (7)	0.0553 (8)	0.0005 (5)	0.0009 (6)	−0.0013 (6)
C10	0.0308 (6)	0.0302 (6)	0.0381 (6)	0.0006 (5)	−0.0001 (5)	−0.0010 (5)
C11	0.0362 (6)	0.0278 (6)	0.0341 (6)	0.0007 (5)	−0.0004 (5)	−0.0010 (5)
C12	0.0318 (6)	0.0300 (6)	0.0324 (6)	0.0028 (5)	−0.0011 (5)	−0.0024 (5)
C13	0.0295 (6)	0.0296 (6)	0.0317 (6)	−0.0005 (5)	0.0002 (4)	−0.0009 (5)
C14	0.0319 (6)	0.0313 (6)	0.0339 (6)	0.0003 (5)	0.0000 (5)	0.0000 (5)
C15	0.0394 (7)	0.0344 (7)	0.0539 (8)	−0.0075 (6)	0.0028 (6)	0.0019 (6)
C16	0.0462 (9)	0.0700 (12)	0.0707 (12)	0.0050 (9)	0.0103 (8)	−0.0242 (10)
C17	0.0386 (8)	0.0397 (8)	0.1037 (15)	0.0030 (7)	−0.0084 (9)	0.0170 (9)
C18	0.0458 (8)	0.0310 (7)	0.0596 (9)	−0.0061 (6)	0.0016 (7)	0.0005 (6)

### Geometric parameters (Å, °)

**Table:**

O1—C8	1.360 (2)	C4—H4A	0.9300
O1—C7	1.448 (3)	C5—C6	1.349 (3)
O2—C8	1.200 (2)	C5—H5A	0.9300
O3—C11	1.3368 (16)	C6—C7	1.506 (3)
O3—C18	1.4452 (18)	C7—H7A	0.9700
O4—C12	1.3408 (16)	C7—H7B	0.9700
O4—H1O4	0.88 (2)	C9—C16	1.529 (2)
O5—C14	1.2149 (17)	C9—C17	1.533 (2)
O6—C14	1.3193 (16)	C9—C10	1.5370 (18)
O6—C15	1.4513 (17)	C11—C12	1.4213 (19)
N1—C8	1.353 (2)	C12—C13	1.3860 (18)
N1—C9	1.4672 (19)	C13—C14	1.4847 (18)
N1—H1N1	0.83 (2)	C15—H15A	0.9600
N2—C11	1.3077 (17)	C15—H15B	0.9600
N2—C10	1.3542 (17)	C15—H15C	0.9600
N3—C10	1.3127 (17)	C16—H16A	0.9600
N3—C13	1.3627 (16)	C16—H16B	0.9600
C1—C6	1.376 (3)	C16—H16C	0.9600
C1—C2	1.414 (5)	C17—H17A	0.9600
C1—H1A	0.9300	C17—H17B	0.9600
C2—C3	1.354 (5)	C17—H17C	0.9600
C2—H2A	0.9300	C18—H18A	0.9600
C3—C4	1.343 (5)	C18—H18B	0.9600
C3—H3A	0.9300	C18—H18C	0.9600
C4—C5	1.375 (4)		
			
C8—O1—C7	117.95 (16)	C17—C9—C10	111.42 (13)
C11—O3—C18	116.35 (11)	N3—C10—N2	126.07 (12)
C12—O4—H1O4	110.4 (15)	N3—C10—C9	119.16 (12)
C14—O6—C15	116.01 (11)	N2—C10—C9	114.70 (12)
C8—N1—C9	121.72 (13)	N2—C11—O3	120.91 (12)
C8—N1—H1N1	117.3 (15)	N2—C11—C12	122.52 (12)
C9—N1—H1N1	114.6 (15)	O3—C11—C12	116.57 (12)
C11—N2—C10	117.20 (12)	O4—C12—C13	127.17 (12)
C10—N3—C13	116.34 (11)	O4—C12—C11	117.66 (12)
C6—C1—C2	119.4 (3)	C13—C12—C11	115.17 (12)
C6—C1—H1A	120.3	N3—C13—C12	122.55 (12)
C2—C1—H1A	120.3	N3—C13—C14	118.93 (11)
C3—C2—C1	118.8 (3)	C12—C13—C14	118.50 (11)
C3—C2—H2A	120.6	O5—C14—O6	123.62 (13)
C1—C2—H2A	120.6	O5—C14—C13	121.63 (12)
C4—C3—C2	121.3 (3)	O6—C14—C13	114.75 (11)
C4—C3—H3A	119.3	O6—C15—H15A	109.5
C2—C3—H3A	119.3	O6—C15—H15B	109.5
C3—C4—C5	119.9 (3)	H15A—C15—H15B	109.5
C3—C4—H4A	120.1	O6—C15—H15C	109.5
C5—C4—H4A	120.1	H15A—C15—H15C	109.5
C6—C5—C4	121.3 (3)	H15B—C15—H15C	109.5
C6—C5—H5A	119.4	C9—C16—H16A	109.5
C4—C5—H5A	119.4	C9—C16—H16B	109.5
C5—C6—C1	119.3 (2)	H16A—C16—H16B	109.5
C5—C6—C7	121.61 (19)	C9—C16—H16C	109.5
C1—C6—C7	119.1 (2)	H16A—C16—H16C	109.5
O1—C7—C6	111.30 (17)	H16B—C16—H16C	109.5
O1—C7—H7A	109.4	C9—C17—H17A	109.5
C6—C7—H7A	109.4	C9—C17—H17B	109.5
O1—C7—H7B	109.4	H17A—C17—H17B	109.5
C6—C7—H7B	109.4	C9—C17—H17C	109.5
H7A—C7—H7B	108.0	H17A—C17—H17C	109.5
O2—C8—N1	126.21 (16)	H17B—C17—H17C	109.5
O2—C8—O1	124.59 (16)	O3—C18—H18A	109.5
N1—C8—O1	109.19 (14)	O3—C18—H18B	109.5
N1—C9—C16	106.99 (13)	H18A—C18—H18B	109.5
N1—C9—C17	109.39 (13)	O3—C18—H18C	109.5
C16—C9—C17	111.45 (16)	H18A—C18—H18C	109.5
N1—C9—C10	109.75 (11)	H18B—C18—H18C	109.5
C16—C9—C10	107.73 (12)		
			
C6—C1—C2—C3	1.6 (5)	C17—C9—C10—N3	−30.53 (19)
C1—C2—C3—C4	−1.8 (6)	N1—C9—C10—N2	31.13 (17)
C2—C3—C4—C5	0.7 (6)	C16—C9—C10—N2	−85.01 (16)
C3—C4—C5—C6	0.6 (5)	C17—C9—C10—N2	152.45 (14)
C4—C5—C6—C1	−0.8 (4)	C10—N2—C11—O3	178.86 (12)
C4—C5—C6—C7	−178.5 (2)	C10—N2—C11—C12	−0.8 (2)
C2—C1—C6—C5	−0.3 (4)	C18—O3—C11—N2	−3.98 (19)
C2—C1—C6—C7	177.4 (3)	C18—O3—C11—C12	175.74 (12)
C8—O1—C7—C6	106.6 (2)	N2—C11—C12—O4	177.56 (12)
C5—C6—C7—O1	−84.3 (3)	O3—C11—C12—O4	−2.16 (18)
C1—C6—C7—O1	98.0 (3)	N2—C11—C12—C13	−2.25 (19)
C9—N1—C8—O2	18.1 (2)	O3—C11—C12—C13	178.04 (12)
C9—N1—C8—O1	−163.27 (13)	C10—N3—C13—C12	−0.14 (19)
C7—O1—C8—O2	−9.7 (2)	C10—N3—C13—C14	−178.53 (11)
C7—O1—C8—N1	171.67 (15)	O4—C12—C13—N3	−177.02 (12)
C8—N1—C9—C16	166.62 (15)	C11—C12—C13—N3	2.76 (19)
C8—N1—C9—C17	−72.52 (18)	O4—C12—C13—C14	1.4 (2)
C8—N1—C9—C10	50.01 (18)	C11—C12—C13—C14	−178.85 (11)
C13—N3—C10—N2	−3.5 (2)	C15—O6—C14—O5	−1.1 (2)
C13—N3—C10—C9	179.89 (12)	C15—O6—C14—C13	179.53 (11)
C11—N2—C10—N3	4.0 (2)	N3—C13—C14—O5	167.78 (13)
C11—N2—C10—C9	−179.24 (12)	C12—C13—C14—O5	−10.7 (2)
N1—C9—C10—N3	−151.85 (13)	N3—C13—C14—O6	−12.83 (17)
C16—C9—C10—N3	92.00 (17)	C12—C13—C14—O6	168.72 (12)

### Hydrogen-bond geometry (Å, °)

**Table:**

*D*—H···*A*	*D*—H	H···*A*	*D*···*A*	*D*—H···*A*
O4—H1O4···O5	0.87 (2)	1.93 (3)	2.6513 (15)	139 (2)
O4—H1O4···O2^i^	0.87 (2)	2.39 (2)	3.0508 (17)	132 (2)
C7—H7A···O5^i^	0.97	2.52	3.347 (3)	143
C15—H15A···O3^ii^	0.96	2.49	3.2148 (18)	132
C17—H17A···O2	0.96	2.54	3.099 (2)	117

Symmetry codes:  (i) −*x*, −*y*+1, −*z*; (ii) *x*, *y*−1, *z*.
